# Innate Lymphoid Cells in Inflammatory Bowel Disease

**DOI:** 10.3390/cells14110825

**Published:** 2025-06-02

**Authors:** Xin Yao, Kaiming Ma, Yangzhuangzhuang Zhu, Siyan Cao

**Affiliations:** Division of Gastroenterology, Washington University School of Medicine, St. Louis, MO 63110, USA; xyao@wustl.edu (X.Y.); ma.k@wustl.edu (K.M.); yangzhuangzhuang@wustl.edu (Y.Z.)

**Keywords:** inflammatory bowel disease, innate lymphoid cells, mucosal immunity, animal models, novel therapies

## Abstract

Inflammatory bowel disease (IBD), including Crohn’s disease and ulcerative colitis, is a chronic inflammatory disorder of the gastrointestinal tract with rising incidence and an unclear etiology. Innate lymphoid cells (ILCs) have recently emerged as key regulators of mucosal immunity and tissue homeostasis and are increasingly implicated in IBD. Unlike adaptive lymphocytes, ILCs do not require antigen recognition and clonal expansion to respond rapidly to environmental cues and shape immune responses. In a healthy gut, ILCs maintain intestinal homeostasis by guarding the epithelial barrier, protecting against pathogens, and mounting proper responses to external insults. However, their altered differentiation, proliferation, recruitment, activation, and interaction with other host cells, microbiota, and environmental stimuli may contribute to IBD. In this review, we discuss recent advances in understanding murine and human ILCs in the context of intestinal inflammation and IBD. A deeper understanding of ILC-mediated immune mechanisms may offer novel therapeutic strategies for restoring intestinal homeostasis and improving personalized management of IBD.

## 1. Overview of IBD

Inflammatory bowel disease (IBD) is a group of chronic inflammatory disorders of the gastrointestinal (GI) tract, mainly comprising Crohn’s disease (CD) and ulcerative colitis (UC). CD is characterized by patchy, transmural lesions that can affect any segment of the GI tract and lead to complications such as fistulas, strictures, and abscesses [1]. UC usually involves continuous, superficial inflammation limited to the colon and rectum with mucosal ulcerations [2]. Chronic inflammation from either UC or CD increases the risk of colorectal dysplasia and carcinoma over time [2,3].

In 2017, the global prevalence was estimated to exceed 6.8 million cases, with the highest age-standardized prevalence rate observed in North America (422 per 100,000; 95% CI: 398.7–446.1) [4]. In the United States alone, approximately 2.39 million individuals have been diagnosed with IBD [5]. The global age-standardized incidence rate (ASIR) of IBD was 4.45 per 100,000 in 2021 [6]. Notably, newly industrialized countries in Asia, Eastern Europe, and Latin America have experienced a particularly rapid increase in IBD incidence [7]. In urban regions of China, the incidence was reported to be 10.04 per 100,000 person-years in 2016 (95% CI: 6.95–13.71) [8]. Both the incidence and prevalence of IBD are projected to continue rising through 2050 [9]. The economic burden associated with IBD is considerable. Recent estimates suggest that the worldwide average direct health care cost is approximately $12,000 for CD and $9000 for UC per patient/year [10].

Despite much progress made during the past 30 years, current therapeutic strategies for IBD remain limited in efficacy and durability. While biologics and small molecules, including anti-TNFα antibodies, anti-interleukin (IL)-23 antibodies, anti-integrin therapies, and Janus kinase (JAK) inhibitors, have tremendously expanded treatment options, their efficacy remains variable among patients [11,12,13]. As a result, the need for more targeted, durable, and personalized therapeutic approaches remains a critical challenge in IBD management [14], which requires a deeper understanding of the complex mechanisms underlying IBD.

Over 300 genetic loci have been implicated in IBD susceptibility [15], with 80 novel loci recently identified in East Asians [16], including NOD2 [17], IL23R [18], and TNFSF15 [17], etc., though the effects of their variants differ across populations [17,19]. Though the etiology of IBD remains unclear, evidence indicates that genetically susceptible individuals exhibit an aberrant immune response to intestinal microbes, coupled with compromised epithelial barrier integrity and skewed immune response [20,21]. This disruption facilitates immune cell infiltration and is associated with dysregulated T helper (Th) cell responses, particularly Th1 and Th17, along with impaired regulatory T cell (Treg) function in CD. These abnormalities drive the overproduction of proinflammatory cytokines such as TNF-α, IFN-γ, IL-1, IL-6, IL-12, and IL-23 [1]. In UC, additional type 2 cytokines, including IL-5, IL-6, and IL-13, also play a key role in shaping the inflammatory microenvironment [2]. This complex cytokine milieu reflects the involvement of both innate and adaptive immune components in IBD.

While T cells have long been recognized as central players in IBD pathogenesis, growing evidence highlights the importance of innate lymphoid cells (ILCs) in orchestrating mucosal immunity over the last 15 years [22]. ILCs share functional parallels with T cells but mainly reside within mucosal tissues, where they can promptly respond to cytokines and other local signals. Lacking antigen-specific receptors, ILCs are critical immune mediators that contribute to mucosal defense, tissue repair, and homeostasis in the gut [23,24]. Given their ability to rapidly respond to environmental cues and influence epithelial integrity and immune balance, ILCs represent a critical but underexplored component in IBD pathophysiology [25]. This underscores the need for a comprehensive review to clarify their contribution to intestinal inflammation and their therapeutic potential in IBD.

## 2. Overview of ILCs

**Figure 1:**
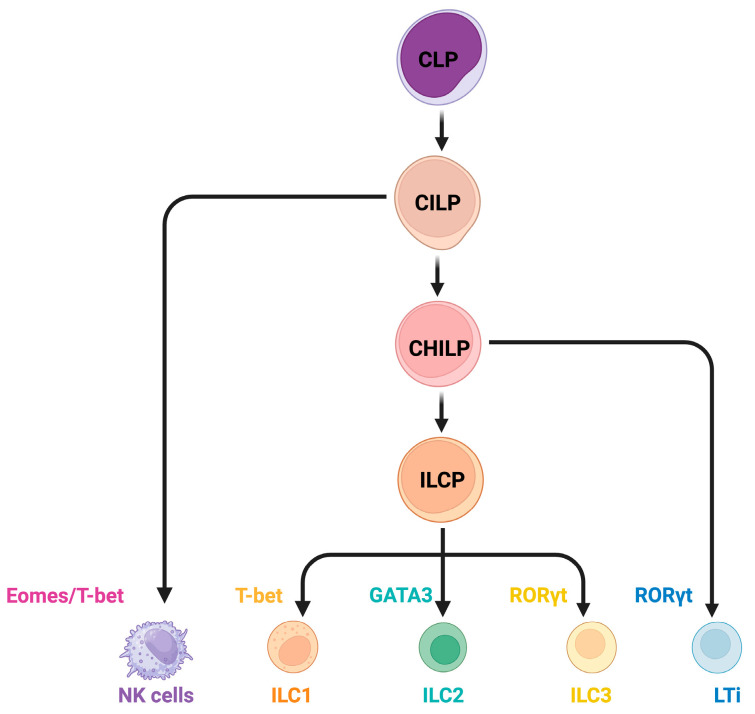
ILC classification and differentiation. In humans and mice, ILCs originate from lymphoid progenitors such as CLPs and LMPPs. Human CLPs initially emerge in the fetal liver before transitioning to the adult bone marrow as the primary site of development. ILC differentiation follows a well-defined hierarchical pathway. CLPs give rise to CILPs, which serve as the earliest precursors within the ILC lineage. CILPs can develop into functional NK cells. Alternatively, CILPs differentiate through the CHILP lineage, giving rise to ILCPs and LTiPs, which further mature into LTis. LTis play a critical role in lymphoid organogenesis, whereas ILCPs differentiate into ILC1s, ILC2s, and ILC3s with distinct functions. Abbreviations: CLP, common lymphoid progenitor; LMPP, lymphoid-primed multipotent progenitor; CILP, common innate lymphoid progenitor; CHILP, common helper-like innate lymphoid progenitor; ILCP, innate lymphoid cell precursor; LTiP, lymphoid tissue inducer progenitor; Eomes, eomesodermin; T-bet, T-box expressed in T cells; GATA3, GATA binding protein 3; RORγt, RAR-related orphan receptor gamma t. Created in BioRender. Cao, S. https://BioRender.com/be3izfi (accessed on 27 May 2025). Used with permission.

In most literature, ILCs are categorized into five subsets—natural killer (NK) cells, lymphoid tissue inducer (LTi) cells, and three groups of helper-like ILCs: ILC1, ILC2, and ILC3 [23] (Figure 1). NK cells parallel CD8^+^ T cells by releasing cytotoxic granules, such as granzyme B and perforin, requiring both transcription factors T-bet and Eomes to differentiate [26,27]. Driven by RORγt, LTi cells play critical roles in embryonic development of secondary lymphoid organs such as lymph nodes and Peyer’s patches [28,29]. Helper-like ILC subsets can be further defined by their transcriptional factors and functional resemblance to CD4^+^ Th cells. ILC1s, regulated by T-bet, produce IFN-γ akin to Th1 cells; ILC2s, under GATA3 control, secrete IL-5 and IL-13, paralleling Th2 cells; and ILC3s, also dependent on RORγt, produce IL-22 and IL-17, similar to Th17 cells [23,30].

In both humans and mice, ILCs are derived from lymphoid progenitors and follow a hierarchical differentiation pathway [23,31]. They progress through stages including common lymphoid progenitors (CLPs), common innate lymphoid progenitors (CILPs), and common helper innate lymphoid progenitors (CHILPs), ultimately giving rise to NK cells, LTi cells, and helper-like ILC subsets. This tightly regulated process, governed by key transcription factors, cytokine signals, and environmental cues, has been well characterized in previous reviews [23,32,33]. In addition to identifying key transcription factors, researchers have developed an in vitro culture system to generate functional human ILCs from CD34⁺ hematopoietic progenitor cells, demonstrating that the combined impact of cytokines and Notch signaling also dictates the differentiation of ILC subsets [34].

Helper-like ILCs are predominantly tissue-resident [35]. The frequency of circulating ILCs accounts for only 0.1% to 1% of total lymphocytes in humans [36]. Within the GI tract, distinct ILC subsets exhibit region-specific localization. In C57BL/6 mice, ILC3s are the most abundant subset in the small intestinal lamina propria (LP), whereas ILC2s are enriched in the colon [37]. Unexpectedly, ILC2s dominated the ILC composition of both the ileum and colon lamina propria in rats [38]. By contrast, the intraepithelial compartment of the small intestine is primarily populated by NK cells and ILC1s [39]. In humans, ILC3s are enriched in the ileum and colon, while ILC1s are predominant in the upper GI tract [40]. In contrast to mice, ILC2s are largely absent from the healthy human colon, as shown by both surface marker analysis and single-cell transcriptomic profiling [41,42]. Moreover, human CD62L⁻ ILCs contain multipotent precursors of ILC1s/NK cells and ILC3s, and exhibit distinct differentiation potentials depending on the tissue microenvironment [43]. The accumulation of CD62L⁻ naïve-like ILCs was also increased in endoscopically inflamed colonic biopsies from pediatric IBD patients, and their frequency positively correlated with disease severity [44]. Because of their mucosal localization and potential for exerting a more localized immunomodulatory effect, ILCs may represent promising therapeutic targets in IBD, offering possibilities for more precise interventions that minimize systemic side effects.

## 3. ILCs in Homeostasis and IBD

**Figure 2:**
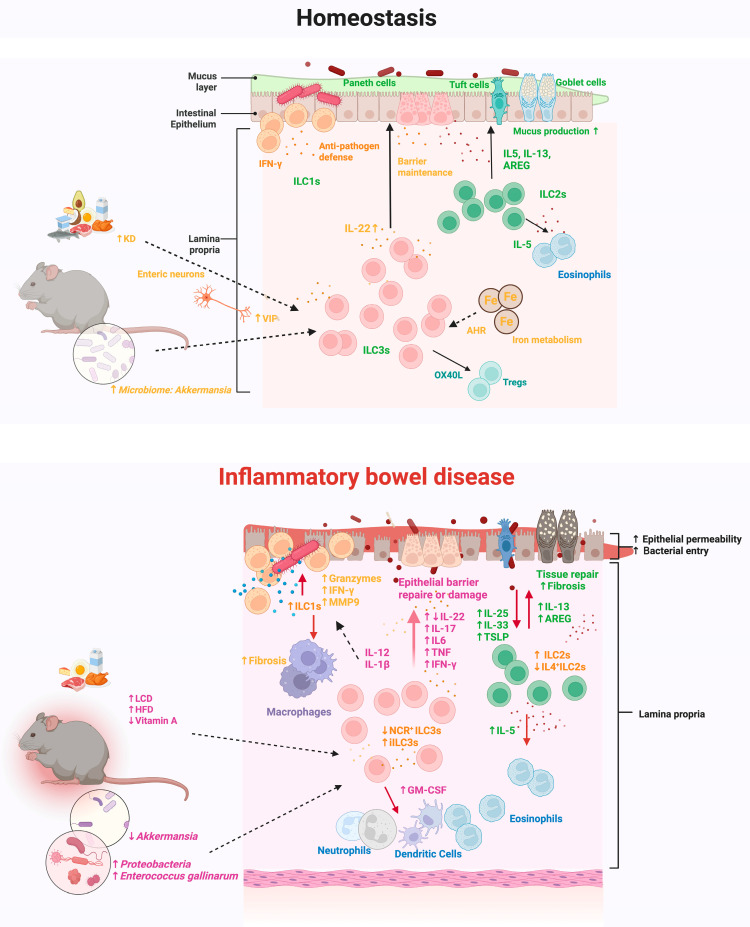
Role of ILCs in intestinal homeostasis and IBD in mouse models. Intestinal ILCs maintain mucosal homeostasis through coordinated interactions with the epithelium, microbiota, dietary factors, and other immune cells. In the healthy gut, ILC1s produce IFN-γ to support host defense against intracellular pathogens. ILC2s secrete IL-5, IL-13, and AREG, promoting mucus production of goblet cells and tissue repair. ILC3s express IL-22, which enhances epithelial barrier function and antimicrobial defense. In the context of IBD, dysregulated ILC responses contribute to epithelial injury and chronic inflammation. ILC1s show increased production of IFN-γ and granzymes, while ILC2 activity becomes exaggerated. ILC3s exhibit functional plasticity and context-dependent roles in IBD, contributing to either mucosal protection or inflammation depending on the local immune milieu. Moreover, as key sensors of dietary stress, ILCs are regulated by multiple environmental cues, including micronutrient availability, dietary components, and changes in the gut microbiota. Abbreviations: ILC, innate lymphoid cell; iILC3: inflammatory ILC3; AHR, aryl hydrocarbon receptor; AREG, amphiregulin; GM-CSF, granulocyte-macrophage colony-stimulating factor; IFN-γ, interferon-gamma; IL, interleukin; MMP9, matrix metalloproteinase 9; OX40L, OX40 ligand; TNF, tumor necrosis factor; TSLP, thymic stromal lymphopoietin; Treg, regulatory T cell; VIP, vasoactive intestinal peptide; KD, ketogenic diet; LCD, low-carbohydrate diet; HFD, high-fat diet; NCR, natural cytotoxicity receptor. Created in BioRender. Cao, S. (2025) https://BioRender.com/wuwrb6n (accessed on 27 May 2025). Used with permission.

The intestinal mucosa functions as a crucial interface between the host and the external environment. Gut homeostasis relies on epithelial barrier function, immune tolerance, and well-controlled inflammatory and wound-healing responses. ILCs help maintain this delicate balance by rapidly responding to local cues, fighting pathogenic invasion, restoring the epithelial barrier, and orchestrating adaptive immune activities. However, when this regulation is disrupted, ILCs can contribute to inflammation and tissue damage, as observed in IBD mouse models (Figure 2).

### 3.1. ILC1

#### 3.1.1. ILC1 in Homeostasis

Under steady state, ILC1s contribute to anti-viral host defense and the maintenance of mucosal homeostasis [45,46,47]. ILC1 subsets include intraepithelial ILC1s (ieILC1s) and lamina propria (LP) ILC1s in both the human and mouse intestine [48]. ieILC1s are characterized by the expression of CD103, an epithelial homing integrin that binds to E-cadherin and likely facilitates their retention within the epithelial layer. These cells exhibit several hallmarks of TGF-β imprinting and display an activated-memory phenotype, producing IFN-γ and lytic mediators in response to IL-12 and IL-15 stimulation [49,50]. ieILC1s also express CD160, which binds to the epithelial cell surface receptor herpes virus entry mediator (HVEM), thereby facilitating their intraepithelial localization and contributing to host defense against acute bacterial infections [51]. LP ILC1s express CD127 and produce IFN-γ in response to stimulation with IL-15 and IL-12 [52,53]. The transcription factor Hobit, encoded by *Zfp683*, has been identified as a key regulator of ILC1 differentiation. In the intestinal mucosa, *Zfp683* expression is highly correlated with ILC1s [54]. Early-stage ILC1s, which already express T-bet but retain gene signatures of ILC progenitors, initiate Hobit expression to guide their stepwise maturation into CD127⁺TCF-1⁺ and, eventually, CD127⁻TCF-1⁻ effector ILC1s [55]. Another distinct subset has been identified in mice, referred to as ex-ILC3 cells. These cells originate from ILC3s that have downregulated RORγt expression and acquired features characteristic of ILC1s [54,56,57]. Intestinal ILC1s from LP expressed high levels of *Cxcr6*, *Ccr9*, *Il7r*, *Tmem176a*, and *Tmem176b* that are also expressed in ILC3s, supporting the notion that a subset of ILC1s may arise through conversion from ILC3s [58].

Serving as frontline defenders in barrier tissues, ILC1s actively surveil the mucosa even under homeostatic conditions. This continuous activity supports antiviral readiness and tissue protection despite the absence of antigen-specific receptors [46]. Additionally, ILC1s exhibited significant developmental potential in the liver, where IFN-γ produced by ILC1s facilitated the proliferation of Lin^−^CD122^+^CD49a^+^ progenitor cells, thereby promoting their own expansion [59].

#### 3.1.2. ILC1 in IBD

Excessive or dysregulated ILC1 activity disrupts epithelial and vascular barriers, thereby aggravating tissue damage [50,60].

Accumulation of both ieILC1s [49] and LP ILC1s [50,53] has been reported in the inflamed ileum of CD patients and the colon of IBD patients [61]. Notably, many of these ILC1s express high levels of granulysin, a cytotoxic molecule implicated in bacterial lysis and monocyte recruitment [52]. A positive correlation has also been observed between the frequency of ILC1s and endoscopic disease severity in CD patients [62]. In addition, circulating ILC1s are elevated in IBD patients [61]; one proposed mechanism for ILC1 expansion is the conversion of ILC3s into ILC1s [63].

The phenotype and function of ILC1s are sculpted by the gut environment. Recent single-cell RNA sequencing (scRNA-seq) of both murine and human tissues indicated that ILC1s can adopt distinct functional states, with certain subsets exerting more pathogenic effects during mucosal inflammation [58,64]. In mice, depletion of ieILC1s by using an anti-NK1.1 antibody reduced intestinal histopathology in an anti-CD40-induced experimental model of innate colitis [49]. Epigenetic studies indicate that ten-eleven translocation (TET) enzymes, which mediate DNA hydroxymethylation, are required for controlling ILC1 proliferation. TET deficiency led to excessive ILC1 expansion, potentially compromising the intestinal barrier [65]. To elucidate the mechanisms underlying ILC1 accumulation in IBD, ILC-intestinal organoid co-culture models were used to explore their functional crosstalk with intestinal epithelial cells. ILC1s isolated from actively inflamed IBD tissues have been shown to secrete TGF-β1 and promote the expansion of CD44v6⁺ epithelial crypts. They also express matrix metalloproteinase 9 (MMP9), which contributes to extracellular matrix (ECM) remodeling and may exacerbate fibrosis and tumor progression under chronic inflammatory conditions [66]. Similarly, ILC1s from the creeping fat of CD patients may also contribute to fibrosis by secreting increased IFN-γ, which in turn stimulates fibrosis-related genes and macrophage inflammatory markers. These ILC1-driven, IFN-γ-mediated pro-fibrotic and pro-inflammatory responses are not only involved in local tissue remodeling but also represent a risk factor for early recurrence following surgery for CD [67].

In summary, ILC1s may contribute to IBD pathogenesis by disrupting intestinal barrier function, promoting chronic inflammation, and driving tissue fibrosis. Targeting their expansion or activation may offer new therapeutic opportunities. However, further studies are needed to dissect the heterogeneity of ILC1 subsets and develop specific interventions to restrain their pathological activity without compromising homeostatic functions such as host defense.

### 3.2. ILC2

#### 3.2.1. ILC2 in Homeostasis

ILC2s play pivotal roles in parasitic and bacterial clearance, allergic reactions, tissue repair, wound healing, and chronic inflammation [68,69]. ILC2s can be categorized into two main subsets: natural ILC2s (nILC2s) and inflammatory ILC2s (iILC2s) in mice [70]. nILC2s are tissue-resident cells characterized by high ST2 (IL-33R) expression, moderate proliferation in response to IL-33 stimulation, and secretion of IL-5 and IL-13 that support local host defense and tissue repair [71]. Conversely, iILC2s express higher levels of KLRG1 and IL-17RB, expand in response to IL-25 stimulation, and can be mobilized from the blood and lymph nodes during inflammation [70]. Human ILC2s were defined by expression of CD127 (the IL-7 receptor α-subunit), CD161 (encoded by *KLRB1*), and CRTH2 (the receptor for prostaglandin D2) [72]. Immature ILC2s can differentiate into IL-5 and IL-13-producing effector ILC2s, including CRTH2⁺CD117⁺ cells that display some ILC3-like features and CRTH2⁺CD117⁻ cells that are more committed to the ILC2 lineage, depending on the tissue microenvironment and cytokine milieu [73,74,75].

Alarmin cytokines IL-25, IL-33, and thymic stromal lymphopoietin (TSLP) provide key activating signals for ILC2s [76,77]. The capacity of alarmins to stimulate ILC2s is age-dependent, with IL-33 playing a central role in ILC2 responses during early life [78]. ILC2s express amphiregulin (AREG) in response to alarmin cytokine stimulation [79,80]. AREG, a member of the epidermal growth factor (EGF) family [81], binds to EGFR to enhance mucin production, thereby supporting mucosal barrier function [71]. Importantly, ILC2-derived AREG played a non-redundant role in tissue protection following intestinal damage and inflammation [82]. Additionally, ILC2s express NMUR1, the receptor for neuromedin U, a cholinergic neuropeptide. ILC2s were rapidly and robustly activated by NMU produced by enteric neurons, leading to the production of IL-5 and IL-13 in an NMUR1-dependent manner [83]. NMUR1^iCre^ mice have been reported to show high specificity for ILC2s [82]. However, a later study revealed that a subset of eosinophils in the small intestine also expresses NMUR1, limiting its exclusivity [84]. The novel model, named Boolean-ILC2-Cre (BIC) mice, was reported to enhance targeting accuracy by using a combinatorial logic based on *Icos*, *Il13*, and *Cd28* expression [85], though it still requires further validation. Similarly, human ILC2s upregulated type 2 cytokine expression upon NMU stimulation ex vivo [86]. ILC2s in the gut express the highest levels of aryl hydrocarbon receptor (AHR) among all ILC subsets [87]. AHR is a ligand-dependent environmental sensor [88] that intrinsically suppresses the expression of the IL-33 receptor ST2 and downregulates ILC2 effector cytokines, thereby serving as a crucial modulator of ILC2 activity [87]. Besides, ILC2s are found to be the predominant source of ILC-derived IL-10 in the mouse intestine [89]. This IL-10⁺ ILC2 subset could upregulate the immunoregulatory checkpoint molecule cytotoxic T-lymphocyte-associated antigen 4 (CTLA-4) expression and contribute to maintaining tissue homeostasis [90].

#### 3.2.2. ILC2 in IBD

In patients with IBD, ILC2s were more frequently observed compared to their near absence in mucosal samples from non-IBD individuals [62], and IL-33 expression was correspondingly increased in the colonic tissue of both IBD patients and dextran sulfate sodium (DSS)-induced colitis [91]. Consistent with these findings, gut mucosal biopsies from patients with ileum-specific CD demonstrated a marked expansion of ILC2s [92]. In patients with active CD, circulating SLAMF1 (signaling lymphocytic activation molecule)-expressing ILC2s inversely correlated with disease severity as measured by the Harvey–Bradshaw Index [93]. In fibrotic CD lesions, *IL-13* transcripts were notably increased, accompanied by infiltration of IL-13Rα1^+^ ILCs [94,95], suggesting a potential role for ILC2s in driving intestinal fibrosis.

In mice, ILC2 expansion occurred early in the intestinal LP of the SAMP1/YitFc model of Crohn’s disease-like ileitis [92]. Mouse-derived ILC2s were expanded in vitro and then transferred to mice with DSS-induced colitis, leading to reduced colonic inflammation [91]. IFN signaling was enriched in ILC2s during DSS-induced colitis, enhancing AREG production and contributing to the protection of epithelial barrier integrity [96]. Furthermore, the Tec family kinase ITK is essential for the survival of ILC2s, and its deficiency led to reduced ILC2 numbers, compromised intestinal barrier function, and increased susceptibility to DSS-induced colitis [69]. Conversely, ILC2s decreased in the terminal ileum of 3-month-old *Tnf*^ΔARE^ mice, a model of spontaneous CD-like ileitis driven by TNF overexpression, suggesting that chronic inflammatory environments may suppress ILC2 responses [97]. Specifically, during inflammation or infection, ILC2s have been reported to acquire notable migratory capacity, which may further exacerbate intestinal inflammation. CCR2⁺ ILC2s could gradually migrate from the lungs to the intestine and adapt to the local microenvironment by transitioning into CCR4⁺ ILC2s [98]. In patients with steroid-resistant asthma, JAK3 inhibitors mitigated disease severity, likely by reducing ILC2 survival, proliferation, and production of IL-5 and IL-13 [99]. Future studies should elucidate the role of ILC2s in steroid-refractory IBD.

Nutrition, stress, and the microbiome are tightly regulated under physiological conditions. These factors are also actively involved in the pathophysiology of IBD [100,101]. A randomized controlled trial showed that inulin supplementation in UC patients increased colonic IL-5 levels and was associated with symptomatic relapse [102]. Dietary inulin elevated microbiota-derived bile acids, which induced IL-33 expression and promoted the expansion of inflammatory ILC2s that preferentially secrete IL-5 but not AREG, leading to eosinophil accumulation and exacerbation of DSS-induced colitis in mice [103]. Malnutrition is common in IBD patients [104]. Dietary vitamin B1 is essential for the maintenance of intestinal tuft cells. Vitamin B1 deficiency reduced tuft cell-derived IL-25, resulting in decreased IL-4⁺ ILC2s, impaired goblet cell differentiation, and aggravated murine colitis induced by 2,4,6-trinitrobenzene sulfonic acid (TNBS) [105].

Additionally, imbalanced interactions between ILC2s and other immune cells could disrupt intestinal immune tolerance. It has been reported that pulmonary ILC2s regulate the balance between local and systemic immunity by secreting leukemia inhibitory factor (LIF) to promote homing of CCR7⁺ immune cells to lymph nodes [106]. Pulmonary ILC2s also enhanced neutrophil infiltration via lipid droplet formation [107] and expanded GATA3^high^ Tregs to suppress Th2 cell activation [108]. Those findings highlighted the immunomodulatory role of ILC2s in inflammation. In the gut, T cells and B cells regulate ILC2 homeostasis by expressing members of the SLAM family, specifically SLAMF3 (CD229) and SLAMF5 (CD84). In mouse models, the deletion of *Slamf3* and *Slamf5* in T and B cells led to an increase in ILC2 numbers and exacerbated inflammatory responses [109].

Taken together, ILC2s have been linked to both reparative and pathogenic roles in IBD. However, current evidence is limited by the specificity of ILC2-targeting tools and the clinical relevance of murine models used in most studies. Further research is needed to further define the regulatory mechanisms in ILC2s and evaluate their potential as biomarkers and therapeutic targets in human IBD.

### 3.3. ILC3

#### 3.3.1. ILC3 in Homeostasis

ILC3s are essential for maintaining mucosal barrier integrity, shaping the microbiota, and regulating adaptive immunity [110,111,112]. ILC3s are classified into three distinct subsets based on the expression of the chemokine receptor CCR6 and the NK cell receptor (NCR), NKp46 in both mice and humans, and NKp44 in humans [113]: NCR⁺ ILC3, CCR6⁺ ILC3, and double-negative (DN) ILC3. The CCR6⁺ subset, also known as adult LTi-like ILC3, is characterized by its phenotypic resemblance to fetal LTi, and is capable of producing IL-22, IL-17A/F, and lymphotoxins [28,114,115,116]. Meanwhile, NCR⁺ ILC3 predominantly produces IL-22 and GM-CSF [117]. Recent findings indicate that a tissue-resident ILC precursor in the small intestinal LP can locally generate ILC3s without continuous bone marrow input, underscoring the plasticity of these cells [118]. Specifically, gut ILC3s highly expressed the thymocyte selection-associated high mobility group box protein 2 (Tox2), which is critical for gut ILC3 maintenance and function [119].

IL-22 is essential for ILC3s in preserving intestinal homeostasis [120,121,122] and is known to be crucially dependent on RORγt, RORα [57], and AHR [123,124]. IL-22 upregulated the expression of IL-18, which in turn augmented the production of antimicrobial factors by Paneth cells and the proliferation of Lgr5⁺ stem cells, further enhancing protective effects [125]. In human small intestinal organoid (hSIO) cultures, IL-22 was vital for Paneth cell formation, the primary source of intestinal antimicrobial peptides (AMPs) [126]. The production of ILC3s-derived IL-22 is regulated by several signals. Among ILC3 subsets, CCR6⁺ LTi-like ILC3s strongly express the dendritic cell (DC)–associated transcription factor ZBTB46 and represent a major source of IL-22 [127]. The p38α-eIF6-Nsun2 axis facilitated rapid production of IL-22 by ILC3s in the gut, thereby enhancing epithelial protection [128]. Loss of hypoxia-inducible transcription factors (HIF-1α) in NKp46^+^ cells increased the expression of IL-22-inducible genes and conferred protection against intestinal damage [129].

Additionally, diet constitutes a highly diverse source of antigens. Even prior to food intake, neuroimmune regulation enables ILC3s to remain in a primed state, allowing them to initiate a faster and more effective immune response during the early phase of pathogen invasion, independent of direct pathogen-induced activation. Vasoactive intestinal peptide (VIP) is strongly induced by feeding and reduced by fasting, activates the secretion of IL-22 in ILC3 through VIPR2, contributing to the maintenance of intestinal epithelial barrier integrity [130,131,132]. Interestingly, upon exposure to pathogens or commensal microbes, ILC3s were shown to acquire “trained immunity” and undergo sustained metabolic reprogramming that enhanced IL-22 production and protective responses upon reinfection [133]. Moreover, ILC3s promote epithelial integrity through IL-22-independent mechanisms, including activation of Hippo-Yap1 signaling in intestinal crypt cells [134] and secretion of the EGFR ligand HB-EGF (heparin-binding EGF-like growth factor), which supports epithelial repair and protection during inflammation [135].

Despite its essential role in mucosal protection, ILC3 activity needs to be tightly regulated to avoid excessive immune activation. Several mechanisms have been identified that limit excessive ILC3 activation. Promyelocytic leukemia zinc finger (PLZF), which is transiently expressed in ILC precursors [136], intrinsically repressed IL-22 production in mature intestinal ILC3s and served as an essential regulator of ILC3 homeostasis [137]. Similarly, non-coding RNAs, such as circular RNA circKcnt2, limited excessive ILC3 activation and prevented inflammatory damage [138].

Beyond producing cytokines, ILC3s contribute to intestinal homeostasis through direct interactions with adaptive immune cells. In particular, they support the expansion and maintenance of peripherally derived Tregs (pTregs), which are essential for sustaining immune tolerance to dietary and microbial antigens [139,140]. NCR⁻ ILC3s in the intestine highly expressed OX40L (encoded by *Tnfsf4*), one of the members of the TNF superfamily. OX40 signaling has been reported to be critical for the development and maintenance of Tregs [141]. Consistently, ILC3s lacking OX40L failed to effectively promote Treg expansion [142]. The identity of antigen-presenting cells (APCs) responsible for inducing pTregs in response to dietary and microbial antigens remains under debate. Proposed APC candidates include ILC3s and other rare subsets such as Thetis cells and Janus cells, which exhibit hybrid features of medullary thymic epithelial cells, DCs, and ILC3s [143,144,145,146]. LTi-like ILC3s presented antigens to pTreg cells via MHCII, which delivered a tolerogenic signal to suppress pro-inflammatory effector T cell responses against the microbiota [32,147,148]. However, a recent study by Rodrigues et al. used novel genetic models to compare *Rorc_E_+7kb^Δ/Δ^* mice deficient in both RORγt⁺ DCs and ILC3s with *Serinc2*^iCre^*Rorc*^fl/fl^ mice lacking ILC3s but not DCs, indicating that ILC3s may not be strictly required for the induction or maintenance of pTreg-mediated immune tolerance [149]. These results highlight the complexity of intestinal immune regulation and underscore the need for further studies that more specifically target ILC3s or RORγt⁺ DCs, along with more rigorous functional validation using physiologic disease models, to clarify their respective roles in pTreg induction and the establishment of mucosal tolerance. Future studies should also define the clinical significance of ILC3s in infections and autoimmune conditions.

#### 3.3.2. ILC3s in IBD

NKp44⁺ ILC3s are consistently and markedly decreased in the ileum of patients with CD and in the colon of patients with UC [61,62,150], and their frequencies negatively correlated with both histological and endoscopic disease severity scores in pediatric IBD [44]. Additionally, HB-EGF–producing ILC3s, which help safeguard epithelial cells from death, were diminished in inflamed IBD tissues [135]. ILC3s from patients with CD closely resembled fetal NCR^+^ ILC3s, suggesting that the CD pathogenesis involves aberrant reactivation of fetal lymphoid organogenesis programs in adulthood [151].

In mice, altered ILC3 function is associated with experimental colitis. ILC3s from inflamed UC tissues exhibited increased expression of Wnt pathway genes [152,153]; similarly, activation of Wnt signaling in murine ILC3s led to downregulation of cell proliferation-related genes, leading to heightened susceptibility to DSS-induced colitis [153]. ILC3s also regulated IL-23-driven Th17 responses through the CTLA-4 signaling pathway, thereby alleviating chronic colitis in mice [154,155]. In addition, TNF abrogated IL-22-mediated mucosal repair during T cell transfer colitis [156]. ILC3-specific deficiency of nucleophosmin 1 (NPM1) in mice did not affect the number of ILC3s but resulted in decreased IL-22 production and exacerbated colitis [157].

Notably, the impact of ILC3s on intestinal inflammation varies depending on the immune compartment and specific context [158]. Intestinal ILC3s express high levels of the transmembrane protein neuropilin-1 (NRP1), which is upregulated in the intestinal mucosa of IBD patients. Genetic ablation of *Nrp1* resulted in reduced ILC3 frequency and IL-17A production, thereby ameliorating DSS-induced colitis [159]. Deficiency of the basic leucine zipper transcription factor ATF-like (BATF) elevated the total ILC3 population in the small intestine, accompanied by a reduction in NCR⁻ ILC3s. This imbalance led to spontaneous colitis, characterized by epithelial disruption, immune cell infiltration, and formation of crypt abscesses [160]. Moreover, in the absence of adaptive immunity, ILC3s adopted an inflammatory phenotype and produced higher amounts of IL-17, IL-22, GM-CSF, and IFNγ, which further exacerbated colitis [161]. Genetic studies have linked *TNFSF15* polymorphisms and its protein TNF-like ligand 1A (TL1A) with IBD [162]. CX3CR1⁺ mononuclear phagocytes (MNPs) released TL1A, promoted IL-22 production by ILC3s in acute colitis, and facilitated mucosal healing in mice [142,163]. However, in chronic colitis, TL1A played a contrasting role by inducing OX40L expression in MHCII⁺ ILC3s, which in turn contributed to T cell–driven chronic inflammation. Accordingly, ILC3s expressed high levels of death receptor 3 (DR3), which mediates TL1A signaling and enhanced GM-CSF production. Elevated GM-CSF levels further promoted the accumulation of eosinophils, neutrophils, and CD11b⁺CD11c⁺ myeloid cells, ultimately leading to ILC3 depletion from the intestine and exacerbation of colitis [164]. Although IL-22 is often regarded as a protective cytokine in mucosal immunity, studies in several chronic models of IBD have paradoxically demonstrated a pathogenic role for this cytokine [165]. IL-22 may serve as a key regulator of neutrophil recruitment to the colon by modulating the expression of CXC-family chemokines with neutrophil-attracting activity. Notably, elevated expression of IL-22-responsive genes has been associated with resistance to ustekinumab therapy in patients with UC [166].

The functions of ILC3s in IBD are also dynamically regulated by environmental cues and the neuroimmune axis. Gut mucosa is covered by a dense and complex coat of sugar chains, which forms an essential niche for microbiota colonization. In patients with IBD, impaired mucosal N-glycosylation is accompanied by a diminished frequency of intestinal ILC3s. This N-glycan remodeling is associated with the downregulation of ILC3-mediated immune responses, promoting a phenotypic shift toward proinflammatory ILC1s and increased TNF-α production [167]. Dysregulation of these processes may indirectly contribute to the pathogenesis of IBD. Endoplasmic reticulum (ER) stress and genetic variants of X-box binding protein 1 (XBP1) have been linked to human IBD [168]. XBP1, a key regulator of the ER stress response, exhibited rhythmic expression in small intestinal ILC3s in mice. Activation of the IRE1α/XBP1 signaling axis enhanced IL-22 production upon IL-23 stimulation and conferred protection against colitis. Importantly, the frequency of intestinal XBP1s⁺ ILC3s before starting ustekinumab, a non-selective anti-IL-23 antibody, was positively correlated with therapeutic response in CD patients [169]. Conversely, chronic psychological stress promoted ILC3 overactivation via cAMP–FOXO1 axis and contributed to intestinal inflammation [170]. How different environmental stimuli dictate the homeostatic vs. pathogenic role of ILC3s in IBD remains to be elucidated.

ILC3s serve as primary sensors of dietary stress, which influences intestinal inflammation. Vitamin D deficiency has been associated with IBD and correlates with worsened disease activity and a higher risk of intestinal resection [171,172]. The active form of vitamin D selectively suppressed the production of IL-22, IL-17, and GM-CSF by activated intestinal NKp44⁺ ILC3s while enhancing IL-6 secretion, which may contribute to the early recruitment of phagocytic monocytes and neutrophils to the bacterial invasion site during the initial phase of IBD [173]. Vitamin A deficiency led to a dramatic reduction of ILC3s, which impaired immunity against acute bacterial infections [174]. Microbial signals are equally crucial for ILC3 function. Colonization of the GI tract by *Candida tropicalis* modulated vitamin B3 metabolism by promoting the conversion of nicotinamide to nicotinic acid. This metabolic shift enhanced IL-17A and IL-22 production by ILC3s, thereby strengthening the intestinal barrier and relieving the disease in a DSS-induced colitis model [175]. The commensal bacterium *Akkermansia muciniphila* stimulated DCs to produce retinoic acid, thereby reinforcing IL-22-mediated barrier function and mitigating colitis [176]. In addition to vitamins, ketogenic diet and low-carbohydrate diet differentially modulated the composition and function of gut microbiota. Ketogenic diet significantly reduced ILC3 abundance and their proinflammatory cytokine expression, thus alleviating colitis. In contrast, a low-carbohydrate diet substantially downregulated Occludin, ZO-1, and Muc2, leading to weakened intestinal barrier integrity [177]. A recent study showed that mice fed a short course of a high-fat diet developed mild signs of colitis accompanied by impaired IL-22 production by enteric ILC3s [178]. AHR also functions as a key environmental sensor and plays a critical role in regulating ILC3 function [123]. Under iron deficiency, downregulated AHR expression compromised the maintenance of gut-resident ILC3s and caused a significant reduction of IL-22 [179].

Altogether, emerging evidence highlights the multifaceted roles of ILC3s in IBD, ranging from barrier protection to promotion of chronic inflammation. However, these findings are complicated by the preclinical models used, tissue-specific cues, disease stages, and environmental/microbial influences. Subset-specific targeting of ILC3s combined with physiologically relevant IBD models will be essential to resolve conflicting evidence.

### 3.4. NK Cells

#### 3.4.1. NK Cells in Homeostasis

NK cells are key components of innate immunity, known for their ability to target viruses, intracellular pathogens, and tumor cells [49,180]. NK cells are found in peripheral blood, cord blood, bone marrow, spleen, lungs, and throughout the intestinal mucosa. They can be generally divided into CD56^bright^ and CD56^dim^ subsets [181,182], the former being more cytokine-oriented and the latter possessing stronger cytotoxic capabilities. NK cells produce IFN-γ and TNF-α upon activation by cytokines such as IL-15, IL-12, and IL-18 [182,183]. Specifically, IL-15 is essential for the development of NK cells, as NK cell numbers are significantly reduced in mice lacking IL-15 or its receptor IL-15Rα [184].

#### 3.4.2. NK Cells in IBD

IFN-γ-producing CD3^−^CD56^+^ NK cells were more abundant in the intestinal mucosa of CD patients compared to those with UC or healthy controls [185]. In UC patients, intestinal epithelial cells (IECs) exhibited significantly elevated expression of HLA-DP molecules, rendering them targets of NK cells and leading to epithelial injury [186]. In treatment-naïve CD patients, peripheral NK cells showed elevated expression of gut-homing integrins and an increased frequency of degranulation events, which tended to normalize following anti-TNF therapy [187]. Another study found that circulating NK cells from patients with active IBD exhibited reduced IFN-γ production but increased secretion of TNF-α and IL-17A upon ex vivo stimulation [188]. Conventional NK (cNK) cells are characterized by co-expression of T-bet and Eomes and the production of cytotoxic molecules. Depletion of NCR^+^ cNK cells, but not ILC1 or ILC3 subsets, aggravated experimental colitis in mice. This suggests that NCR^+^ cNK cells have a unique and non-redundant protective function against intestinal inflammation [189].

IL-15 promoted NK cell differentiation into the CD56^bright^ subset, augmented their cytotoxic activity [190], and was overexpressed in the inflamed IBD mucosa [191]. Similarly, IL-21 was substantially elevated in the inflamed intestinal mucosa of CD patients [192], and has been shown to induce the activation of NK cells from the peripheral blood of IBD patients in vitro [193]. A recent study demonstrated that anti-Saccharomyces cerevisiae antibodies (ASCAs), which are often present in individuals with CD years before diagnosis, can selectively activate NK cells as evidenced by increased granzyme B secretion and cytotoxic degranulation [194]. These findings suggested that NK cell activation may represent an early immunological event in the preclinical phase of CD.

Taken together, although NK cells are known for their cytotoxic capacities, their roles in IBD remain poorly characterized. Further studies should clarify the distinct roles of NK cells in acute and chronic inflammation in the mouse intestine and assess their contribution to disease onset, remission, and relapse in IBD patients.

## 4. ILCs in IBD Therapies

When pathogenic ILC activities are stimulated and/or protective, the ILC’s function is impaired, and it tends to exacerbate a variety of inflammatory diseases, including IBD [195]. In the following section, we summarize existing evidence linking ILCs to major IBD treatments (Table 1) and explore how this may inform the development of novel therapeutic approaches and personalized management of IBD.

As the oldest category of biological therapy in IBD, anti-TNF drugs indirectly suppress ILC1-mediated inflammation [61]. Vedolizumab is an α4β7 integrin antibody approved for moderately to severely active UC and CD. Treatment with vedolizumab resulted in a decrease in ILC1s and an increase in NCR^+^ ILC3s [61]. IL-23 plays a key role in IBD pathogenesis [200]. IL-23 blockers include ustekinumab, which binds the shared p40 subunit of IL-12 and IL-23, and newer agents, such as risankizumab, guselkumab, and mirikizumab that target the IL-23-specific p19 subunit [201]. ILC3s express IL-23R and respond to IL-23 stimulation by producing cytokines including IL-22 and IL-17, indicating that IL-23 blockers may significantly impact ILC3 function [161]. In addition to IL-23, treatment by anti-IL-12 was shown to induce trans-differentiation of human NCR⁺ ILC3s into an ILC1-like ex-ILC3 phenotype ex vivo [53]. Surprisingly, ustekinumab treatment resulted in a slight but significant increase in the NCR^+^ ILC3 population in the human intestine [61]. The JAK-STAT pathways are closely linked to ILC development, activation, and plasticity [202] by regulating a variety of cellular processes triggered by a variety of inflammatory cytokines [203]. Tofacitinib, a potent oral JAK1/JAK3 inhibitor approved for UC, modulated innate immune response by reducing the frequency of ILC1s and their production of IFN-γ [196].

In addition to approved treatments, preclinical and clinical studies have examined the therapeutic value of targeting ILC-related cytokines with mixed results. Anti-IL-5 therapy showed therapeutic potential in experimental colitis [204]. IL-5 receptor antagonist YM-90709 ameliorated DSS-induced colitis in mice by inhibiting the NLRP3 inflammasome and reducing IL-1β in the colon [205]. Anti-IFN-γ antibody fontolizumab for treating active CD did not exhibit notable clinical effectiveness compared with a placebo control [206]. Neutralizing anti-IL-13 antibodies anrukinzumab and tralokinumab were tested in UC without significant benefit [207,208]. Randomized clinical trials using recombinant human GM-CSF (sargramostim), another ILC3-produced cytokine, failed to improve remission rates in patients with CD [209]. IL-17A inhibition by secukinumab has demonstrated benefits for autoimmune conditions including psoriasis, psoriatic arthritis, axial spondyloarthritis, rheumatoid arthritis, and systemic lupus erythematosus [210]. However, secukinumab treatment led to worsened outcomes in some CD patients; this unexpected outcome was likely due to impaired IL-17R signaling in IECs and disrupted barrier integrity that exacerbated chronic inflammation [211]. IL-22 agonism using efmarodocokin alfa demonstrated safety in a phase Ib trial, yet clinical efficacy data have not been available [212].

The limited success of targeting ILC-related cytokines highlights the complexity of IBD pathogenesis and the need to explore alternative therapeutic avenues. One promising direction involves modulation of ILC migration and tissue distribution. Sphingosine-1-phosphate receptors (S1PRs) are a class of G protein-coupled receptors that bind lipid signaling molecules and orchestrate the migration and distribution of adaptive lymphocytes and ILCs [213]. In IBD, elevated S1P levels were shown to promote migration of inflammatory cells from lymph nodes and infiltration into the intestinal mucosa [214]. ILC2s and ILC3s may migrate between organs in an S1P-dependent manner [215,216]. Fingolimod, an oral S1PR modulator approved for multiple sclerosis, decreased small intestinal ILC3s in mice and suppressed cytokine production by ILC1s and ILC3s in vitro [197]. Selective S1PR1/5 modulator ozanimod is approved for treating moderate to severe UC [217,218], although limited information exists about its impact on ILC populations and trafficking.

Another promising treatment approach involves targeting the TL1A signaling pathway. Anti-TL1A agent tulisokibant showed a favorable safety and efficacy profile to achieve clinical remission for UC in a phase 2 trial [219]. Duvakitug, an anti-TL1A monoclonal antibody, recently demonstrated positive phase 2b results in UC and CD patients “https://www.sanofi.com/en/media-room/press-releases/2024/2024-12-17-12-30-00-2998154” (accessed on 27 May 2025). TL1A is highly expressed in the inflamed intestinal mucosa of IBD patients, while its receptor, DR3, is abundantly expressed on ILCs [220,221,222]. TL1A stimulated the expansion, survival, and activity of ILC2s independent of alarmin cytokines IL-25 and IL-33 [223]. Meanwhile, IL-22 production is upregulated in a DR3-dependent manner in both mouse and human ILC3s [163,224]. The impact of the TL1A-DR3 pathway on ILCs is complex and only partially understood. Future endeavors should focus on how TL1A-DR3 blockade modulates various ILC subsets in preclinical and clinical models of IBD.

With a deeper understanding of mucosal immunity and ILCs in intestinal inflammation, newer therapeutic strategies are being explored for refractory IBD. The immunomodulatory imide lenalidomide was shown to degrade Ikaros and Aiolos, thereby impeding ILC1 differentiation and augmenting ILC3 properties [199]. Thus, lenalidomide may help reverse certain pathogenic phenotypes by altering the plasticity of ILC subpopulations in IBD. Indigo naturalis, an activator of the AHR-IL-22 pathway, demonstrated efficacy in UC patients, with clinical response rates reported between 40% and 94% [225]. However, one multicenter randomized controlled trial was discontinued following the development of pulmonary arterial hypertension in a participant [226]. Approved for psoriasis and atopic dermatitis [227], AHR agonist tapinarof cream downregulated type 2 cytokines IL-4 and IL-13, mitigated oxidative stress, and normalized skin barrier integrity [228]. R848 is a TLR7 agonist that activates CD11c⁺ DCs to secrete IL-23. Upon R848 treatment, IL-22 and IFN-γ expression were specifically elevated in ILCs and promoted intestinal stem cell proliferation [229]. These findings highlight the therapeutic potential of targeting ILCs and related pathways in IBD, although further studies are needed to assess their clinical applicability.

Although ILCs and Th cells share key transcription factors and cytokine profiles, accumulating evidence indicates that ILCs exert non-redundant roles in immune regulation [82,230]. ROR-γt is critical for the development and function of both Th17 cells and ILC3s. Some evidence suggests that Th17 cells are primarily associated with proinflammatory responses, whereas ILC3s are more implicated in tissue homeostasis and repair. Interestingly, ROR-γt inhibition ameliorated colitis in *Il10*^−/−^ mice by reducing Th17 cell frequencies without affecting ILC3 populations in the colon [231]. This strategy holds therapeutic potential by enabling selective suppression of pathogenic Th17 cell activity and upregulation of protective ILC subsets in the context of IBD.

## 5. Challenges and Future Directions in ILCs in IBD

During the past 10–15 years, significant progress has been made in the relevance of ILCs and IBD. While these findings have deepened our understanding of ILC function, several limitations persist.

1. The heterogeneity and plasticity of ILCs add substantial complexity to the studies. Variation in marker selection criteria across studies has led to inconsistencies in the definition of some ILC subsets, making cross-study comparisons challenging.

2. ILC-targeting strategies in mice yet lack subset specificity and may inadvertently affect other immune cells, such as Th cells and myeloid cells.

3. The translational relevance of ILC studies is further hindered by differences between animal models and human disease. The vast majority of mechanistic studies have relied on murine models, yet substantial differences exist between murine and human ILCs in terms of differentiation, distribution, and function.

4. The function of ILCs in IBD complications, including stricturing/fistulizing CD, inflammatory pouch conditions, and IBD-associated malignancies, remains under-investigated.

5. Furthermore, the contribution of ILC plasticity to distinct stages of human IBD (e.g., remission, relapse, progression) remains unclear, as clinical studies primarily rely on cross-sectional analysis of colonic biopsies or peripheral blood, restricting the capacity for dynamic assessment.

6. Therapeutic tools that target ILCs are lacking.

## 6. Conclusions

Taken together, ILCs can exhibit both protective and deleterious functions in the gut, and display context-dependent effects in acute versus chronic inflammation, underscoring the need for carefully calibrated interventions. Novel targets hold the promise of expanding treatment options, particularly for patients with refractory disease.

## Figures and Tables

**Table 1:** Impacts of current and investigational IBD treatments on human and mouse ILCs.

	Treatments	Mechanism	Effects on Humans or Mice	Circulating or Tissue-Resident	Impacts on ILCs	
					Increased	Decreased
**Approved**	Infliximab or Adalimumab [61]	anti-TNFα	Humans	Intestinal	NCR^+^ ILC3s	ILC1s
				Circulating	NCR^-^ ILC3s	ILC1s
	Vedolizumab [61]	anti-α4β7 integrin	Humans	Intestinal	NCR^+^ILC3s	ILC1s
				Circulating	NCR^−^ ILC3	-
	Ustekinumab [61]	anti-IL-12/IL-23	Humans	Intestinal	NCR^+^ ILC3s	NCR^-^ ILC3
				Circulating	ILC1s	-
	Tofacitinib [196] (rheumatoid arthritis)	JAK1/JAK3 inhibitor	Humans	Circulating	-	IFN-γ^+^ ILC1s
	Fingolimod [197] (multiple sclerosis)	S1PR modulator	Mice	Intestinal	-	ILC2s and ILC3s
				Circulating	-	Total ILCs
			Humans	Tonsillar	-	IFN-γ^+^ ILC1s GM-CSF^+^ ILC3s
**Clinical trials**	DR3-Fc [165]	Anti-TL1A	Mice	Intestinal	Restored ILC3s	GM-CSF^+^ ILC3s
	Indigo naturalis [198]	AHR agonist	Mice	Intestinal	NK cells	ILC2s and ILC3s
	Lenalidomide [199]	Degrades Ikaros and Aiolos	Mice	Tonsillar	Restored ILC3s	ILC1s and NK cells

## Data Availability

No new data were created or analyzed in this study. Data sharing is not applicable to this article.
